# Prevalence and determinants of unintended pregnancy in sub-Saharan Africa: A multi-country analysis of demographic and health surveys

**DOI:** 10.1371/journal.pone.0220970

**Published:** 2019-08-09

**Authors:** Edward Kwabena Ameyaw, Eugene Budu, Francis Sambah, Linus Baatiema, Francis Appiah, Abdul-Aziz Seidu, Bright Opoku Ahinkorah

**Affiliations:** 1 The Australian Centre for Public and Population Health Research, Faculty of Health, University of Technology Sydney, Sydney, NSW, Australia; 2 Department of Population and Health, College of Humanities and Legal Studies, University of Cape Coast, Cape Coast, Ghana; 3 Department of Health, Physical Education, and Recreation, University of Cape Coast, Cape Coast, Ghana; Anglia Ruskin University, UNITED KINGDOM

## Abstract

**Introduction:**

Approximately 14 million unintended pregnancies are recorded annually in sub-Saharan Africa (SSA). We sought to investigate the prevalence and determinants of unintended pregnancies among women in sub-Saharan Africa.

**Materials and methods:**

The study pooled data from current Demographic and Health Surveys (DHS) conducted from January 1, 2010 to December 31, 2016 from 29 countries in SSA. Logistic regression analysis was used to examine the factors that influence unintended pregnancies in SSA. Results were presented using odds ratios (OR).

**Results:**

We found overall unintended pregnancy prevalence rate of 29%, ranging from 10.8% in Nigeria to 54.5% in Namibia. As compared to women aged 15–19 years, women of all other age categories had higher odds of unintended pregnancies. Married women were 6 times more probable to report unintended pregnancy as compared to women who had never married (OR = 6.29, CI = 5.65–7.01). The phenomenon had higher odds among rural residents as compared to urban residents (OR = 1.08, CI = 1.01–1.16). Women with primary (OR = 0.74, CI = 0.69–0.80) and secondary (OR = 0.71, CI = 0.65–0.77) levels of education had less chances of unintended pregnancies, compared to those with no education. Again, women in all other wealth categories had less probability of unintended pregnancy, as compared to women with poorest wealth status.

**Conclusion:**

Our study contributes substantially towards the discourse of maternal wellbeing by unveiling the prevalence and determinants of unintended pregnancy across the SSA region. There is the need for SSA countries with high prevalence of unintended pregnancies to consider past and present successful interventions of other countries within the region such as health education, counselling, skills-building, comprehensive sex education and access to contraception. Much of these efforts rest with the governments of SSA countries.

## Introduction

Unintended pregnancy accounted for 44% of all global pregnancies between 2010 and 2014 [1] and this makes up 62 unintended pregnancies per 1,000 women aged 15–44 years. A significant proportion of these pregnancies result in abortion and other adverse pregnancy outcomes [1, 2]. Unintended pregnancy is a pregnancy that occurs either when no child or children are desired (unwanted) or when it was not expected (mistimed) [3]. It is usually an outcome of nonuse, inconsistent use or incorrect use of effective family planning methods [3]. Globally, there has been a decline in unintended pregnancy but this has been uneven between the high-income countries, compared with low- and middle-income countries, where 65 unintended pregnancies per 1,000 women aged 15–44 (30%) and 45 per 1,000 women (16%) decline respectively occurred between 2010 and 2014 [1]. In spite of this general decline and the widespread availability of various family planning methods, the phenomenon remains high in sub-Saharan Africa (SSA) [4].

Approximately 14 million unintended pregnancies are recorded annually in sub-Saharan Africa [5]. Some studies have revealed complex underpinnings influencing unintended pregnancies in various pathways [6–8]. These include poor knowledge in contraceptive use and low socio-economic status [9], contraceptive failure [6], sexual violence [7, 8], shortage in contraceptive supply [6], coerced contraceptive decision-making [8], unmarried status and other socio-demographics [6]. Other findings from some countries within the sub-region have attributed the current unintended pregnancy situation to inconsistent and incorrect condom use, contraceptive failure, and lack of knowledge on emergency contraception [10–12].

Unintended pregnancy predisposes women to several risk factors such as unsafe abortion, maternal death, malnutrition, mental illness and vertical transmission of HIV to children [13–15]. Hubacher et al. [5] also indicated that the risk of unintended pregnancy in SSA continues to be high and unsafe, and this predisposes approximately 1 in 16 women to psychosocial impacts of morbidity and mortality [16]. It increases stress levels, impacts negatively on women’s quality of life, and threatens economic status of families [17, 18].

Studies that have investigated predictors of unintended pregnancy in SSA were mainly country-specific, with the focus on Ghana [19, 20], South Africa [12, 21], Kenya [22, 23], Nigeria [24], Ivory Coast [25] and Ethiopia [26, 27]. Our extensive search indicated that no effort has been made to investigate the phenomenon across the SSA region. As most unintended pregnancies occur in the low- and middle-income countries, including SSA countries [28], there is a critical need to investigate the underlying factors for unintended pregnancies among women in SSA. The outcome of this multi-country study will improve reproductive wellbeing of women by unveiling the underlying factors that must be targeted by governments of SSA countries and reproductive health-focused organisations that operate in the sub-region.

## Materials and methods

### Data source

The study made use of pooled data from current Demographic and Health Surveys (DHS) conducted from January 1, 2010 and December 31, 2016 in 29 countries in sub-Saharan Africa. The countries are Angola, Benin, Burkina Faso, Burundi, Congo DR, Congo, Côte d’Ivoire, Cameroon, Chad, Comoros, Ethiopia, Gabon, Ghana, Gambia, Guinea, Kenya, Liberia, Lesotho, Mali, Malawi, Namibia, Nigeria, Rwanda, Sierra Leone, Senegal, Togo, Uganda, Zambia and Zimbabwe. These 29 countries were included in the study because they had current DHS data and also all the variables of interest for this study. Our study included these 29 countries under the DHS program in order to provide a holistic and in-depth evidence of unintended pregnancy in SSA. DHS is a nationwide survey collected every five-year period across low- and middle-income countries. The survey is representative of each of these countries. Women’s files were used for our study and these files possess the responses by women aged 15 to 49. The survey targets core maternal and child health indicators such as unintended pregnancy, contraceptive use, skilled birth attendance, immunisation among under-fives and intimate partner violence.

The DHS survey employs stratified two-stage sampling technique in order to ensure national representativeness [29]. As described in detail previously [30] the first-stage constituted the development of a sampling frame consisting of a list of primary sampling units (PSUs) or enumeration areas (EAs) which cover the entire country and are usually developed from the available latest national census. Each PSU or EA is further subdivided into standard size segments of about 100–500 households per segment. In this stage, a sample of predetermined segments is selected randomly with probability proportional to the EA’s measure of size (number of households in EA).

In the second stage, DHS survey personnel select households systematically from a list of previously enumerated households in each selected EA segment, and in-person interviews are conducted in selected households with target populations: women aged 15–49 and men aged 15–64. The number of selected households per EA is variable and ranges from 30 to 40 households/women per rural cluster and from 20 to 25 households/women per urban cluster [30]. The surveys were done in different times due to the variations in the starting points of the DHS in the various countries. The sample frame usually excludes nomadic and institutional groups such as prisoners and hotel occupants. As evidence in other studies combining the DHS in sub-Saharan Africa [30–32], although the starting points of the data surveys are different, this does not defeat the ability to compare the DHS among the countries. Permission to use the data set was sought from MEASURE DHS. The data set is available to the public at https://dhsprogram.com/data/available-datasets.cfm.

### Definition of variables

#### Dependent variable

The dependent variable for the study was “pregnancy intentions” which arose from the question regarding whether women wanted their current pregnancy or not. It had three responses: ‘then’, ‘later’ and ‘not at all’. Following the definition of unintended pregnancy as “pregnancies that are either wanted earlier or later than occurred (mistimed) or not needed (unwanted)” (CDC, 2015) [3], we coded these three responses as follows: then = 0 ‘intended’; ‘later and not at all’ = 1 ‘unintended’. The inclusion criteria was all women (15–49) who had answered this particular question.

#### Explanatory variables

Eleven explanatory variables were considered in our study. These are age, marriage, place of residence, wealth, parity, occupation, education, religion, contraceptive use intention, knowledge of contraception and country of origin. Apart from country of origin, the rest of the variables were not determined *a priori*; instead, the selection was based on their significant association with the outcome variable, unintended pregnancy. Additionally, a number of these variables have been reported as predictors of unintended pregnancies [6–8, 19, 20]. Six of these variables were recoded to make them meaningful for analysis and interpretation. Marriage was recoded into ‘never married (0)’, ‘married (1)’, ‘cohabiting (2)’, ‘widowed (3)’ and ‘divorced (4)’. Occupation was captured as ‘not working (0)’, ‘managerial (1)’, ‘clerical (2)’, ‘sales (3)’, ‘agricultural (4)’, ‘household (5)’, ‘services (6)’ and ‘manual (7)’. We recoded parity (birth order) as ‘zero birth (0)’, ‘one birth (1)’, ‘two births (2)’, ‘three births (3)’, and four or more births (4)’. We recoded religion as ‘Christianity (1)’, ‘Islam (2)’, ‘Traditionalist (3)’, and ‘no religion (4)’. Contraceptive knowledge was recoded as ‘knows no method (0)’, ‘knows traditional (1)’, and ‘knows modern (2)’. Finally, intention of contraceptive use was recoded into ‘intends to use (1)’, and ‘does not intend to use (2)’.

### Statistical analysis

The analysis began with computation of unintended pregnancy prevalence among the 29 SSA countries. Secondly, we appended the dataset and this generated a total sample of 36,529. After appending, we calculated the overall prevalence and proportions of unintended pregnancy across the socio-demographic characteristics with their significance levels and chi-square (χ2) values. Logistic regression analysis was carried out in a hierarchical order where the first model (Model I) was a bivariate analysis of the effect of country on unintended pregnancies. Angola was chosen as the reference country because previous studies have identified no contraceptive use [33–35], and high unmet need for family planning [34, 36] in the country. In Model II, we adjusted for the effect of the other explanatory variables to ascertain how these variables induce unintended pregnancies using a multivariate analysis. The choice of reference categories for these explanatory variables was similarly informed by propositions of some previous studies [5, 6, 37]. Logistic regression was employed because our dependent variable (unintended pregnancy) was measured as a binary factor. Results for the regression analysis have been presented as odds ratios (OR), with their corresponding 95% confidence intervals (CI) signifying precision and significance of the reported OR. Any OR less than one (1) denotes less odds of unintended pregnancy whereas those higher than one (1) indicate higher odds of unintended pregnancy. The inherent sample weight was applied and all analyses were carried out with STATA version 13.0.

### Ethical approval

The DHS surveys obtain ethical clearance from the Ethics Committee of ORC Macro Inc. as well as Ethics Boards of partner organisations of the various countries such as the Ministries of Health. During each of the surveys, either written or verbal consent was provided by the women. Since the data was not collected by the authors of this paper, we sought permission from MEASURE DHS website and access to the data was provided after our intent for the request was assessed and approved on 27^th^ January, 2019.

## Results

### Descriptive results

**Fig 1:**
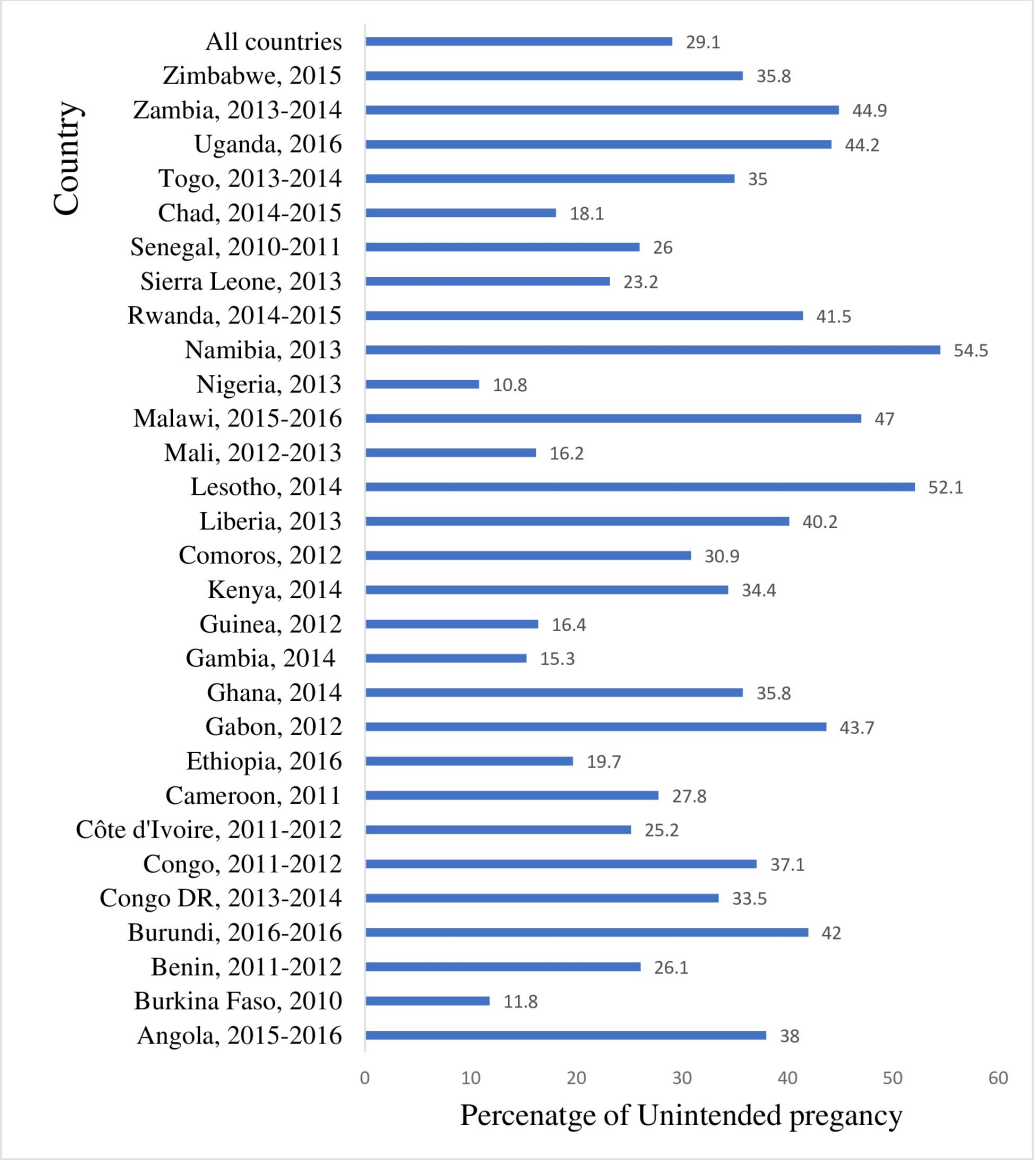
Prevalence of unintended pregnancy in SSA.

In Fig 1, we present the prevalence of unintended pregnancies in each of the 29 SSA countries included in the study. We found that the prevalence of unintended pregnancies ranged from 10.8% in Nigeria to 54.5% in Namibia. Overall, the prevalence of unintended pregnancies in the 29 SSA countries was 29.0%.

Table 1 summarizes the proportion of unintended pregnancies across the included socio-demographic characteristics. Women in the 40–44 age category had a greater proportion of unintended pregnancies (37.8%) whilst the least proportion was recorded among women aged 25–29 (25.3). More than half of pregnancies occurring among never married women were unintended (62.3%) whilst only 22.8% were recorded among the married. Urban residents had 30.5% of unintended pregnancies whereas 28.4% of unintended pregnancies occurred among the women in rural areas. Approximately thirty-seven percent (37.3%) of unintended pregnancies occurred among women with primary level of education, whilst women with higher educational level reported the lowest prevalence (19.0%). About three in ten (30.7%) of unintended pregnancies occurred among women with middle and richer wealth index. Women with four or more births reported 33.6% of unintended pregnancies while women who have had one birth reported 24.9% unintended pregnancies.

**Table 1:** Relationship between socio-demographic variables and unintended pregnancy.

Variables	Weighted n = 36,529	Weighted %	Intended Pregnancy	Unintended Pregnancy	χ2 (P-value)
**Age**					307.4 (P<0.001)
15–19	5,499	15.1	63.3	36.7	
20–24	9,348	25.6	72.8	27.2	
25–29	9,287	25.4	74.7	25.3	
30–34	6,762	18.5	72.6	27.4	
35–39	3,957	10.8	69.0	31.0	
40–44	1,409	3.9	62.2	37.8	
45–49	269	0.7	68.2	31.8	
**Marital status**					9.5 (P<0.001)
Never married	2,718	7.4	37.7	62.3	
Married	26,011	71.2	77.2	22.8	
Cohabiting	6,777	18.6	63.8	36.2	
Widowed	145	0.4	53.0	47.0	
Divorced	879	2.4	46.5	53.5	
**Residence**					16.6 (P<0.001)
Urban	12,014	32.9	69.5	30.5	
Rural	24,514	67.1	71.6	28.4	
**Educational level**					6.3 (P<0.000)
No education	14,451	39.6	80.1	19.9	
Primary	11,683	32.0	62.7	37.3	
Secondary	9,165	25.1	65.1	34.9	
Higher	1,230	3.4	81.0	19.0	
**Wealth index**					51.9 (P<0.001)
Poorest	7,819	21.4	72.5	27.5	
Poorer	8000	21.9	70.1	29.9	
Middle	7,373	20.2	69.3	30.7	
Richer	7,112	19.5	69.3	30.7	
Richest	6,225	17.0	73.7	26.3	
**Parity**					202.7 (P<0.001)
Zero birth	7,683	21.0	70.5	29.5	
One birth	6,910	18.9	75.1	24.9	
Two births	5,885	16.1	73.7	26.3	
Three births	4,875	13.4	73.0	27.0	
Four or more births	11,176	30.6	66.4	33.6	
**Occupation**					134.0 (P<0.001)
Not working	12,695	34.8	71.0	29.0	
Managerial	1,112	3.1	77.2	22.8	
Clerical	199	0.55	81.7	18.3	
Sales	6,856	18.8	73.8	26.2	
Agricultural	11,472	31.4	69.0	31.0	
Services	1,778	4.9	68.1	31.9	
Manual	2,417	6.6		6.6	
**Religion**					7.1 (P<0.001)
Christianity	21,560	59.0	63.5	36.5	
Islam	12,970	35.5	83.1	16.9	
Traditionalist	1,197	3.3	70.8	29.2	
No religion	802	2.2	68.0	32.0	
**Intention to use contraceptive**					6.5 (P<0.001)
Intend to use	21,391	58.6	63.4	36.6	
Does not intend to use	15,137	41.4	80.8	19.2	
**Knowledge on contraceptives**					382.8 (P<0.001)
Knows no method	2,912	8.0	85.0	15.0	
Knows traditional method	281	0.8	84.0	16.0	
Knows modern method	33,337	91.3	69.4	30.6	

Women who were Clerics had the lowest proportion of unintended pregnancies (18.3%). Christian women recorded 36.5% of unintended pregnancies whereas only 16.9% happened among Muslim women. Unintended pregnancy stood at 36.6% among women who had the intention to use contraceptives and 30.6% for women who knew modern contraceptive methods. A smaller proportion (15%) of women who had no knowledge of contraception had unintended pregnancies. Results of the bivariate analysis show that all the socio-demographic variables had a statistically significant relationship with unintended pregnancy at 95% confidence interval (see Table 1).

### Logistic regression results

Table 2 shows the outcome of the Logistic Regression analysis. In Model 1, the 29 SSA countries were considered without the socio-demographic characteristics. With Angola as the reference country, the highest odds of unintended pregnancies was recorded among women of Nigeria (OR = 5.08, CI = 4.39–5.87), followed by Burkina Faso (OR = 4.58, CI = 3.82–5.51). Namibia had less odds of unintended pregnancies, as compared to Angola (OR = 0.51, CI = 0.42–0.63). After adjusting for the socio-demographic variables in Model II, higher odds of unintended pregnancy was still occurring among women of Nigeria (OR = 2.28, CI = 1.92–2.71) and Burkina Faso (OR = 2.36, CI = 1.91–2.92), compared to women of Angola. However, women of Lesotho had less odds of unintended pregnancies (OR = 0.36, CI = 0.22–0.58). As compared to women aged 15–19 years, women of all other age categories had higher odds of unintended pregnancies. Married women were 6 times more probable to report unintended pregnancy, as compared to women who had never married (OR = 6.29, CI = 5.65–7.01).

**Table 2:** Logistic regression on determinants of unintended pregnancy in SSA.

Variables	Sample Size	Model I Odds Ratio (OR) 95% CI	Model II Odds Ratio (OR) 95% CI
**Country**			
Angola	1,404	Ref	Ref
Burkina Faso	1,648	4.58^***^[3.82–5.51]	2.36^***^[1.91–2.92]
Benin	1,492	1.74^***^[1.49–2.04]	0.78^**^[0.65–0.94]
Burundi	1,339	0.85^*^[0.73–0.99]	0.52^***^[0.44–0.63]
Congo DR	2,360	1.22^**^[1.07–1.40]	0.85^*^[0.73–0.99]
Congo	1,093	1.04[0.89–1.23]	1.16[0.96–1.41]
Côte d'Ivoire	993	1.82^***^[1.53–2.18]	1.18[0.96–1.44]
Cameroon	1,438	1.60^***^[1.37–1.88]	1.20^*^[1.01–1.43]
Ethiopia	1,104	2.51^***^[2.09–3.01]	1.11[0.90–1.37]
Gabon	851	0.79^**^[0.67–0.94]	1.04[0.85–1.26]
Ghana	679	1.10[0.91–1.33]	0.70^***^[0.55–0.84]
Gambia	812	3.37^***^[2.71–4.20]	1.19[0.93–1.52]
Guinea	958	3.11^***^[2.54–3.80]	1.13[0.90–1.42]
Kenya	964	1.17[0.99–1.39]	0.80^*^[0.66–0.97]
Comoros	323	1.38^*^[1.06–1.78]	0.46^***^[0.35–0.62]
Liberia	826	0.91[0.77–1.09]	0.96[0.78–1.16]
Lesotho	92	0.64^*^[0.42–0.98]	0.36^***^[0.22–0.58]
Mali	960	3.19^***^[2.60–3.91]	1.22[0.96–1.55]
Malawi	1,828	0.69^***^[0.60–0.80]	0.47^***^[0.40–0.56]
Nigeria	4,198	5.08^***^[4.39–5.87]	2.28^***^[1.92–2.71]
Namibia	527	0.51^***^[0.42–0.63]	0.84[0.67–1.06]
Rwanda	949	0.87[0.73–1.03]	0.59^***^[0.48–0.71]
Sierra Leone	1,373	2.03^***^[1.72–2.39]	1.16[0.95–1.43]
Senegal	1,292	1.75^***^[1.48–2.06]	0.60^***^[0.49–0.73]
Chad	2,382	2.76^***^[2.38–3.21]	1.13[0.95–1.35]
Togo	803	1.14[0.95–1.37]	0.42^***^[0.35–0.52]
Uganda	1,856	0.77^***^[0.67–0.89]	0.71^***^[0.61–0.844]
Zambia	1,379	0.75^***^[0.65–0.87]	0.70^***^[0.58–0.83]
Zimbabwe	606	1.10[0.90–1.34]	0.82[0.65–1.02]
**Age**			
15–19			Ref
20–24			2.00^***^[1.82–2.19]
25–29			2.99^***^[2.68–3.34]
30–34			3.71^***^[3.28–4.20]
35–39			3.43^***^[3.00–3.93]
40–44			2.41^***^[2.04–2.84]
45–49			3.04^***^[2.29–4.05]
**Marital status**			
Never married			Ref
Married			6.29^***^[5.65–7.01]
Cohabiting			4.23^***^[3.79–4.73]
Widowed			3.20^***^[2.24–4.57]
Divorced			2.10^***^[1.77–2.50]
**Residence**			
Urban			Ref
Rural			1.08^*^[1.01–1.16]
**Educational level**			
No education			Ref
Primary			0.74^***^[0.69–0.80]
Secondary			0.71^***^[0.65–0.77]
Higher			0.86[0.70–1.05]
**Wealth index**			
Poorest			Ref
Poorer			0.88^***^[0.82–0.95]
Middle			0.85^***^[0.79–0.92]
Richer			0.82^***^[0.76–0.90]
Richest			0.94[0.84–1.05]
**Parity**			
Zero birth			Ref
One birth			0.61^***^[0.56–0.67]
Two births			0.36^***^[0.33–0.41]
Three births			0.27^***^[0.24–0.31]
Four or more births			0.14^***^[0.13–0.16]
**Occupation**			
Not working			Ref
Managerial			1.48^***^[1.24–1.77]
Clerical			2.24^***^[1.52–3.32]
Sales			1.19^***^[1.10–1.28]
Agricultural			1.19^***^[1.11–1.28]
Services			1.03[0.90–1.17]
Manual			1.06[0.95–1.19]
**Religion**			
Christianity			Ref
Islam			1.43^***^[1.32–1.55]
Traditionalist			1.04[0.89–1.21]
No religion			1.03[0.87–1.22]
**Intention to use contraceptives**			
Intend to use			Ref
Does not intend to use			1.87^***^[1.76–1.99]
**Knowledge on contraceptives**			
Knows no method			Ref
Knows traditional method			1.08[0.77–1.52]
Knows modern method			0.74^***^[0.66–0.83]

Exponentiated coefficients; 95% confidence intervals in square brackets

* p<0.05

** p<0.01

*** p<0.001; Ref = Reference

Higher odds occurred among rural residents as compared to urban residents (OR = 1.08, CI = 1.01–1.16). Women with primary (OR = 0.74, CI = 0.69–0.80) and secondary (OR = 0.71, CI = 0.65–0.77) levels of education had less odds of unintended pregnancies, compared to those with no education. Again, women in all wealth categories had less odds of unintended pregnancy, as compared to poorest women. The phenomenon had less odds among every woman who had ever given birth. However, the odds decreased with increasing parity. With occupation, women who were engaged in managerial (OR = 1.48, CI = 1.24–1.77), clerical (OR = 2.24, CI = 1.52–3.32), sales (OR = 1.19, CI = 1.10–1.28) and agriculture (OR = 1.19, CI = 1.11–1.28) were more probable to experience unintended pregnancies, as compared to non-working women. Muslim women had higher odds ratio of unintended pregnancies, as compared to Christians (OR = 1.43, CI = 1.32–1.55). Women without contraception use intention had 1.87 (CI = 1.76–1.99) odds of having unintended pregnancies. We found that women who knew modern contraceptive method had less odds of unintended pregnancy (OR = 0.74, CI = 0.66–0.83), compared to those who knew no method of contraception (see Table 2).

## Discussion

The greatest proportion of unintended pregnancies occur among women in the low- and middle-income countries, most of which end up in life threatening clandestine-induced abortions [1]. Since nearly all SSA countries fall within the low- and middle-income status [28], this study investigated the current situation of unintended pregnancy in order to direct policies and interventions to key indicators stimulating this burden.

We realised that unintended pregnancy ranges between 10.8% (Nigeria) and 54.5% (Namibia) in SSA. This indicates a wider unintended pregnancy range for SSA than the 20–40% range earlier reported [5]. It is of essence to appreciate that, inasmuch as unintended pregnancies are high in SSA, some significant variations exist. The multivariate analysis revealed that women from Nigeria have higher chances of experiencing unintended pregnancies, compared to women in Angola. A nation’s ability to achieve around 10% unintended pregnancies as realised in Nigeria is an indication of some well-founded and effective operational reproductive health education and contraceptive advocacy policies/interventions. For instance, Nigeria is one of the 41 countries to commit to the FP2020 (Family Planning 2020) [38], and has since 2012 aimed at strengthening family planning service integration at all levels of care as reflected in its national blue print scale-up plan. This is a strategic approach to halting unintended pregnancy and its associated maternal and newborn adverse outcomes [39].

In the case of Namibia, family planning services have been the core in maternal and child health service provision even prior to the emergence of the “reproductive health” as a concept [40]. However, there is limitation in the service, especially for adolescents and men [40]. Mere existence of any health service or intervention does not guarantee utilisation whilst utilisation does not necessarily translate into expected outcome as purported by the framework for evaluation of quality in care in maternity services [41].

Inasmuch as the recent Namibian Health Policy (2010–2020) acknowledged maternal health as a public health concern, nothing was indicated about pregnancy intentions and possible outcomes [42]. The unequal prevalence of unintended pregnancy cannot be well explored without considering the political terrain in which it occurs. In the view of Hulton et al. [41], health service can produce quality outcome if political zeal exists. Unfortunately, a considerable number of countries in the SSA have experienced episodes of unstable governance due to wars and *coup d’états* which possibly drift the attention of some nations from concentrating efforts on critical public health concerns like unintended pregnancy and its resultant adverse outcomes. For instance, within 46 years (1956–2001), 80 successful and 108 unsuccessful *coup d’états* occurred within the sub-region [43] with a significant proportion of them occurring after 2010 [44].

Our study also found that women in other age categories were more likely to experience unintended pregnancies, compared to women aged 15–19 years. This finding is in line with a study by Hubacher, Mavranezouli and McGinn [5], who investigated the magnitude of unintended pregnancies in sub-Saharan Africa and the potential role of contraceptive implants to alleviate it, and found that women aged 20–24, 25–29 and 30–34 years have the highest proportion of unintended pregnancies, compared to those aged 15–19 years. Calvert et al. [45] also reporting from Tanzania indicated that the risk of unplanned pregnancies increases with age. The findings, however, contradict some country-level studies which found a negative relationship between age and unintended pregnancy [19, 20, 46]. This finding could be attributed to the fact that adult women might already have the desirable number of children and, as such, consider any additional pregnancy as unwanted.

Unintended pregnancies were low among women who were unmarried. This finding on marital status and unintended pregnancies confirm the findings of Nyarko [20], who explained that the higher risk of unintended pregnancy among married women may be due to non-use of contraceptives or contraceptive failure, as many married women may have the belief that contraceptive use has serious side effects while some may also believe that it is a sin and hence would have unprotected sex resulting in higher chances of unintended pregnancies.

We noticed that unintended pregnancy occurring among women in rural settings was as twice what their urban counterparts were experiencing. Consistent evidence from the SSA region have similarly indicated that rural women stand greater risks of unintended pregnancies [47–49]. Unintended pregnancy among rural residents call for greater shifts in family planning services in SSA because such women have high chances of delaying ANC, a situation that puts the woman and fetus at greater risks [50]. One reason for the rural-urban differences in unintended pregnancies could be that, in rural areas, there is a high possibility of lack of access to contraceptives compared to urban areas. Even where there is access to contraceptives, limitations such as socio-cultural norms and poor spousal communication may exist [9]. Again, women in rural areas may have low socio-economic status, compared to those in urban areas. These rural residents may, therefore, perceive a pregnancy as unintended if they feel that they cannot cater for the unborn child with their current socio-economic standing.

The observation that poorest women bear higher share of unintended pregnancy is supported by some previous studies in low- and middle-income countries [37, 51], where the situation is reported to be relatively high when compared to the high-income countries [4]. This could indicate their inability to afford modern contraceptives whilst at the same time either not following or being failed by natural birth control methods. If any of these explanations suffice this finding, then it is not surprising if they resort to “cheap” (with quack medical practitioners) abortions where higher complications have been reported [52]. To some extent, we may side with the Theory of Reason Action that, as rational human beings, women would determine the net consequence of seeking pregnancy service (including abortion) with quack medical practitioners before embarking on it [53], yet not having money to access quality care could force women to the quack medical practitioners without a reflective and thorough assessment of the merits and demerits.

The least proportion of unintended pregnancy was borne by women with higher level of education and women with higher wealth status. Similar findings have been reported from high income countries (including U.S.A. and Britain) and among young unmarried women in Israel Military and other parts of the world [54–56]. Education is conceived by the Ecological Model as a powerful individual level predictor of human actions [57]; it has the potential to raise women’s consciousness about the implications of unintended pregnancies and possible contraceptive methods which educated women are probably taking advantage of.

Lamina [24] espoused a statistically significant association between level of education and wealth and use of a method to avoid or delay pregnancy. Hence, women who can make decisions on the use of pregnancy prevention methods will be in a better position of preventing unintended pregnancies. Again, uneducated women may be ignorant or may have limited depth of knowledge about contraceptives and their reproductive system as means of preventing unintended pregnancies [19]. Nyarko [20] also explained that Ghanaian women with some formal education or who have high wealth status are more empowered to take charge of their sexual and reproductive health matters than women who have no formal education or who have poorer wealth status.

The study indicated that almost four of ten women who had four or more births experienced unintended pregnancy. Significant downward total fertility rate is occurring around the globe, of which SSA is no exception [58]. Possibly, women in the region are becoming increasingly conscious of benefits of having small family size in the low- and middle-income countries where high population growth abounds [59]. Women who were not working recorded the highest prevalence, as compared with women who were working in all various occupation categories. This could be that women who are working are much conscious about pregnancies due to their work demands, possible limited maternity leave periods and other disincentives.

We noted that women who did not intend to use contraception were more likely to experience unintended pregnancy. This supports studies by Roy et al. [60] and Abraha, Belay and Welay [61] who found that by expressing the intention to use contraception, women are able to better visualize their future need and are more likely to translate it in to actual practice. The possible reason for this finding could be linked to the relationship between intention to use contraceptives and use of contraceptives. Hence, having no intention to use contraception implies that these women are less likely to use contraceptives and have higher chances of experiencing unintended pregnancies. We also found that the likelihood of unintended pregnancies decreased with knowledge on modern contraceptives. The relationship between modern contraceptive knowledge and unintended pregnancy has been explained in several studies. For instance, Little, Griffin, Dickson and Sadler [62] identified that poor modern contraceptive knowledge is an important area for intervention in primary care since modern contraceptive knowledge is one of the protective factors for unwanted pregnancy. Similarly, Wong, Atefi, Majid and Su [63] found that female participants who had experienced an unplanned pregnancy had a significantly lower modern contraceptive knowledge. These findings have important implications for the development of effective sexual and reproductive educational programmes among this population.

### Strengths and limitations

This study is backed by national representative surveys from 29 countries under the DHS program. The inclusion of only variables with significant association with unintended pregnancy fortifies the rigor of our models, thereby making the findings and conclusions more reliable and replicable to other regions of the world. In spite of the acknowledged strengths, caution needs to be applied in interpreting the results because the study followed a cross-sectional design which does not permit the establishment of causality of unintended pregnancy in SSA.

## Conclusion

Our study has contributed substantially towards the discourse of maternal wellbeing by unveiling the prevalence and determinants of unintended pregnancy across the SSA region. In SSA, the determinants of unintended pregnancy are age, marital status, place of residence, education, wealth quintile, parity, occupation, religion, contraceptive use intention and knowledge on contraception. Directing regional and national level family planning and maternal wellbeing policies and interventions to poor women, women residing in rural settings, the married, those in the mid reproductive age group (25–39) can greatly contribute towards reversing the current dynamics of unintended pregnancy in the SSA region. Again, there is the need to encourage women to use modern methods of contraception in order to reduce their likelihood of experiencing unintended pregnancies. There is the need for SSA countries with high prevalence of unintended pregnancies to consider past and present successful interventions of other countries within the region. Some of these interventions are health education, counselling, skills-building, comprehensive sex education and access to contraception. Much of these efforts rest with the governments of SSA countries because national agenda cannot coincide with these anti-unintended pregnancy interventions without consistent political will.
